# Statistical Analysis of Stress Signals from Bridge Monitoring by FBG System

**DOI:** 10.3390/s18020491

**Published:** 2018-02-07

**Authors:** Xiao-Wei Ye, You-Hua Su, Pei-Sen Xi

**Affiliations:** Department of Civil Engineering, Zhejiang University, Hangzhou 310058, China; ceyhsu@zju.edu.cn (Y.H.S.); cepsxi@zju.edu.cn (P.S.X.)

**Keywords:** structural health monitoring, orthotropic steel bridge, FBG sensor, wavelet multi-resolution analysis, finite mixture distribution, genetic algorithm, Bayesian information criterion, stress concentration factor

## Abstract

In this paper, a fiber Bragg grating (FBG)-based stress monitoring system instrumented on an orthotropic steel deck arch bridge is demonstrated. The FBG sensors are installed at two types of critical fatigue-prone welded joints to measure the strain and temperature signals. A total of 64 FBG sensors are deployed around the rib-to-deck and rib-to-diagram areas at the mid-span and quarter-span of the investigated orthotropic steel bridge. The local stress behaviors caused by the highway loading and temperature effect during the construction and operation periods are presented with the aid of a wavelet multi-resolution analysis approach. In addition, the multi-modal characteristic of the rainflow counted stress spectrum is modeled by the method of finite mixture distribution together with a genetic algorithm (GA)-based parameter estimation approach. The optimal probability distribution of the stress spectrum is determined by use of Bayesian information criterion (BIC). Furthermore, the hot spot stress of the welded joint is calculated by an extrapolation method recommended in the specification of International Institute of Welding (IIW). The stochastic characteristic of stress concentration factor (SCF) of the concerned welded joint is addressed. The proposed FBG-based stress monitoring system and probabilistic stress evaluation methods can provide an effective tool for structural monitoring and condition assessment of orthotropic steel bridges.

## 1. Introduction

During the past three decades, increasing attentions within the engineering and academic communities have been paid to the technology of structural health monitoring (SHM) for the sake of tracking the environmental loadings and structural behaviors in a continuous and real-time manner [1]. The advances of large-scale SHM systems provide effective measures for engineering structures to operate in the safety range [2,3,4]. Meanwhile, with the development of innovative measurement, transmission and signal processing technologies, the SHM systems for various types of civil infrastructures are instrumented to detect the structural damage, evaluate the structural safety condition and predict the remaining service life [5,6]. At present, a considerable number of SHM systems have been installed on large-scale engineering structures worldwide [7,8,9,10,11]. The global or local structural monitoring data of target engineering structures can be achieved, which will greatly facilitate the assessment of structural safety conditions and establishment of inspection and maintenance strategies.

The available practical experiences have proved that the progressive advancement of sensing techniques will undoubtedly expedite the evolution of the SHM technology. In comparison with the traditional mechanical and electrical sensors, the optical fiber sensors possess some unique advantages such as small size, light weight, immunity to electromagnetic interference (EMI) and corrosion and embedding capability. Therefore, amongst the advanced sensing approaches developed in the field of SHM, the optical fiber sensing technology has gained a growing number of attentions by the researchers and engineers [12,13]. The monitoring items can be readily related to the optical characteristics such as change of light intensity, interferometry, fiber Bragg grating (FBG), absorption, time domain reflectometry and frequency domain reflectometry. Instrumentation of bridges using diversified optical fiber sensors are widely reported in the literatures [14,15,16,17,18,19,20]. With the aid of the FBG sensing technology, a growing number of investigators have paid attentions to structural safety assessment of in-service bridges [21,22,23,24,25,26]. Also, investigations have been carried out on monitoring of dynamic responses and damage of structures based on FBG sensors [27,28,29,30].

In this study, an FBG-based stress monitoring system instrumented on an orthotropic steel bridge is first introduced. The FBG sensors are deployed on the fatigue-prone rib-to-deck and rib-to-diagram welded joints at the mid-span and quarter-span of the bridge. The vehicle-induced and temperature-induced stress components in the vicinity of welded joints during the construction and operation periods are derived with the aid of a wavelet multi-resolution analysis approach. In addition, the multiple statistical characteristic of stress spectrum is modeled by the method of finite mixture distribution and a proposed genetic algorithm (GA)-based parameter estimation approach. The optimal distribution of the mixed distribution is achieved by Bayesian information criterion (BIC). Furthermore, the hot spot stress of the concerned welded joint is calculated with an extrapolation method recommended in the specification of International Institute of Welding (IIW) [31] and the stochastic property of stress concentration factor (SCF) is also addressed.

## 2. Analysis of Stress Monitoring Data

### 2.1. FBG-Based SHM System

**Figure 1:**
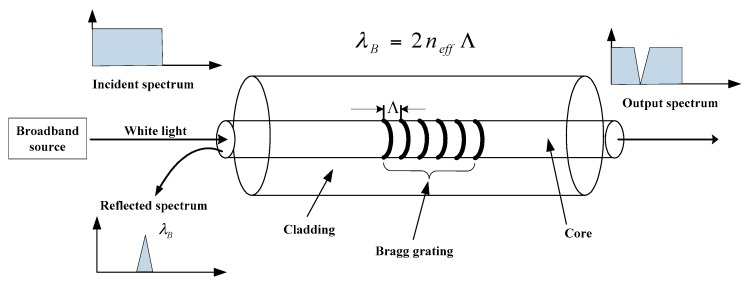
Measurement principle of FBG sensor.

Generally, an optical fiber sensor system consists of a light transmitter, a receiver, an optical fiber, a modulator element and a signal processing unit. As the core part of an optical fiber sensor, the optical fiber itself can act as a sensing element or carry the light from the source to the modulator element, which is usually made from silica glass or polymer material. In accordance with the change of grating spacing, the optical fiber sensor modulates the light and reflects back an optical signal to the analytical unit for deriving the concerned physical quantity of the structure [5]. Up to now, the FBG sensor has been widely used in monitoring of civil engineering structures. It can be regarded as a type of optical fiber sensor with varied refractive indices in the core. According to the Bragg’s law, a beam of white light is written in the FBG sensor and when the light from the broadband source passes through the grating at a particular wavelength, the Bragg wavelength *λ_B_* is reflected, which is related to the effective index of refraction *n_eff_* and the grating period Λ, as illustrated in Figure 1.

It is difficult to determine the stress distribution for the welded joints of steel bridges in comparison with the structures which are mainly composed by relative simple bars or beams due to the complexity of welded joints of the longitudinal and transverse plate-type structural members at their intersections. However, this issue can be tackled with an instrumented SHM system since the monitoring strain data can be directly obtained through the deployed sensors in the vicinity of the welded details. In general, the experimental stress analysis is accomplished based on the strain measurements using the electric resistance strain gauges. In comparison with the traditional mechanical and electrical sensors, the optical fiber sensors possess some unique advantages and therefore they have been employed in monitoring of engineering structures worldwide.

**Figure 2:**
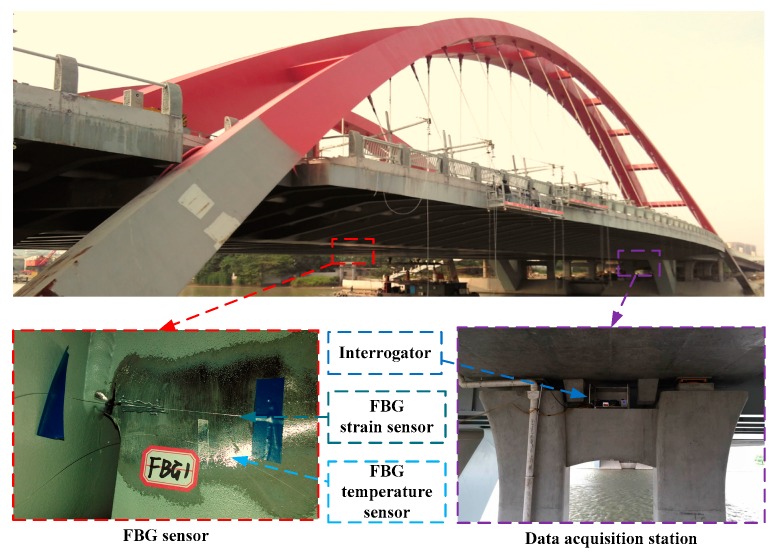
Deployment of FBG-based SHM system.

**Figure 3:**
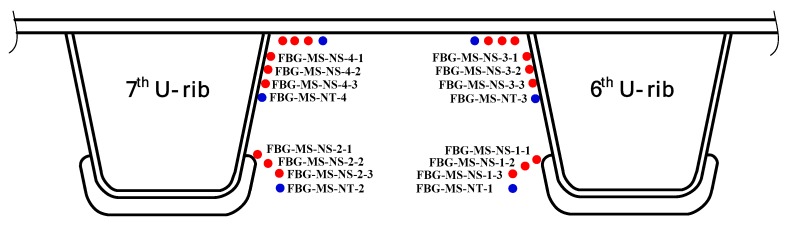
Deployment of FBG sensors.

**Figure 4:**
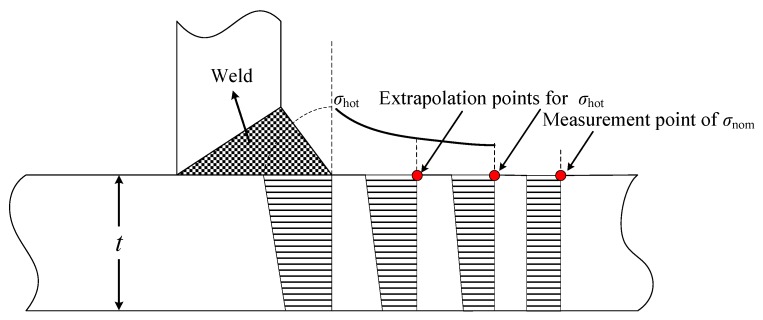
Locations of measurement points.

**Figure 5:**
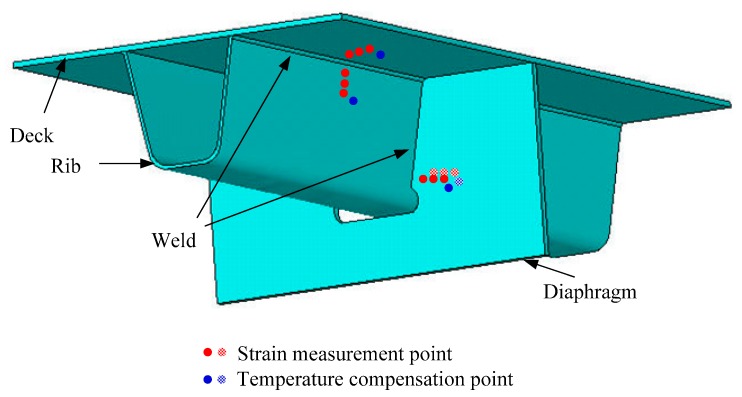
Details of measurement points.

In this study, an FBG-based SHM system was instrumented on a steel bridge crossing the Beijing-Hangzhou Grand Canal located in Hangzhou, China as shown in Figure 2. This system is mainly comprised of FBG sensors, transmission fibers and an interrogator. A total of 16 measurement regions (for each region, it consists of three strain measurement points and one temperature compensation point) are selected for sensor deployment on the critical welded joints at the mid-span and quarter-span sections. The selection of welded joints in each section is primarily based on the literature such as the fatigue-prone rib-to-deck and rib-to-diaphragm with cutout welded joints. The installation locations of the FBG sensors are illustrated in Figure 3, Figure 4 and Figure 5. In detail, four FBG sensors are installed on each measurement area of the 6th and 7th U-rib in the orthotropic steel deck at the mid-span and quarter-span of the bridge. In this study, the measurement areas are relative narrow and we need to install three FBG sensors in a length of 15 mm to capture the stress values at the locations of 5 mm, 15 mm and 20 mm away from the weld toe. The short grating region will cause weak reflected light. In recognition of this, the FBG sensors with a grating region of 1 mm long are customized and the effectiveness the FBG sensors is verified by the sensor supplier. According to the test report, the reflectivity of these FBG sensors is more than 85%. The reflection center wavelengths of the FBG sensors are ranged from 1526 nm to 1561 nm. The interrogator with 16 channels is used in this study and four FBG sensors are linked to each channel.

### 2.2. Data Analysis

For an instrumented SHM system, the effectiveness of the raw monitoring data should be examined and verified according to the data variation tendency. In this study, the FBG-based system was implemented during the bridge construction period and therefore the raw data of each FBG sensor during the bridge construction and operation periods can be obtained. The FBG temperature sensor numbered FBG-MS-NT-2 and the FBG strain sensor numbered FBG-MS-NS-2-3 are selected herewith to perform the original data analysis and comparative study. The selected two sensors are located in the vicinity of the welded joint of the 7th U-rib to diaphragm at the mid-span of the investigated steel bridge. In particular, the sensor FBG-MS-NS-2-3 is located at the position with a distance of 15 mm away from the welded joint and the sensor FBG-MS-NT-2 is close to the sensor FBG-MS-NS-2-3, as illustrated in Figure 3.

**Figure 6:**
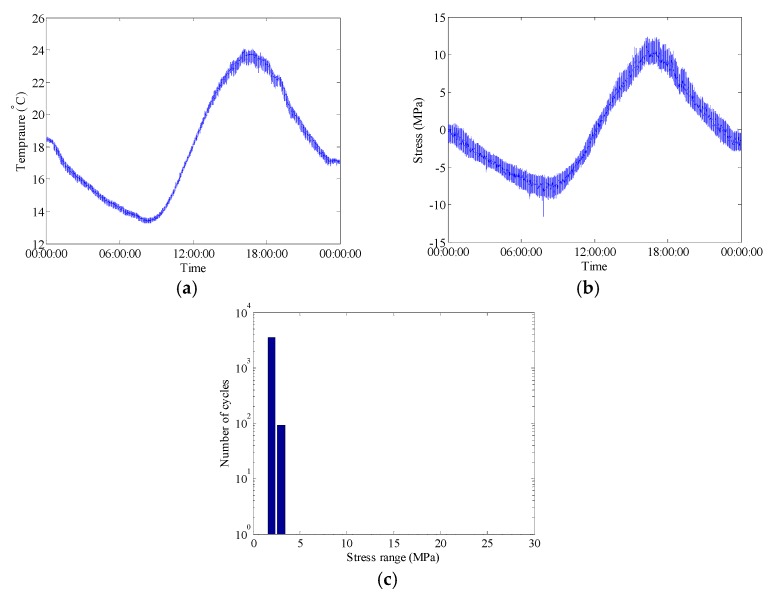
Raw data of selected FBG sensors during bridge construction phase on 17 October 2015: (**a**) Temperature time history measured by FBG-MS-NT-2; (**b**) Stress time history measured by FBG-MS-NS-2-3; (**c**) Histogram of daily stress spectrum.

**Figure 7:**
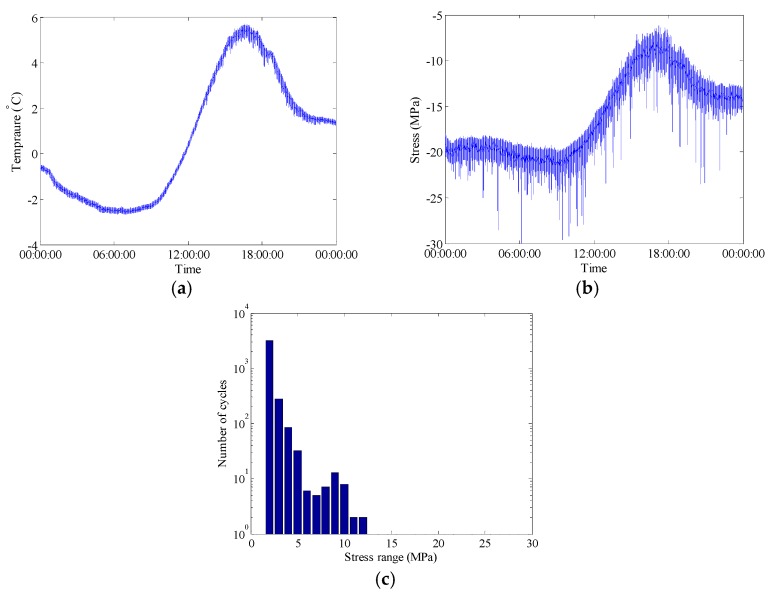
Raw data of selected FBG sensors during bridge operation phase on 29 December 2015: (**a**) Temperature time history measured by FBG-MS-NT-2; (**b**) Stress time history measured by FBG-MS-NS-2-3; (**c**) Histogram of daily stress spectrum.

The variation of the Bragg wavelength Δ*λ_B_* can be expressed as [32]:

(1) $$\Delta \lambda_{B}=\lambda_{B}\{(\alpha +\xi )\Delta T+(1-p_{e})\Delta \varepsilon \}$$

where Δ*ε* is the strain variation, Δ*T* is the temperature change, *α* is the coefficient of the thermal expansion, *ξ* is the thermo-optic coefficient and *p_e_* is the strain-optic coefficient. In this study, according to the coefficients given in the sensor test report and the variation of the Bragg wavelength recorded by the interrogator, the temperature change and the strain variation can be obtained. The raw data of the selected FBG sensors during bridge construction phase on 17 October 2015 is shown in Figure 6. The temperature of steel bridge surface is fluctuated approximately from 13 °C to 24 °C and the stress variation tendency is quite similar with that of the surface temperature. It can be concluded that temperature-induced stress takes a large part of the total stress. In addition, the stress spectrum is derived from the stress time history using the rainflow counting method [33]. During the bridge construction period, the stress ranges of the selected welded joint are less than 5 MPa, which are mainly caused by ambient dynamic loads. Similarly, during bridge operation period, the stress variation tendency is also quite similar with that of the surface temperature as shown in the Figure 7. The stress spectrum for bridge operation period exhibits a multi-modal characteristic containing the stress components induced by ambient dynamic loads and highway traffic.

**Figure 8:**
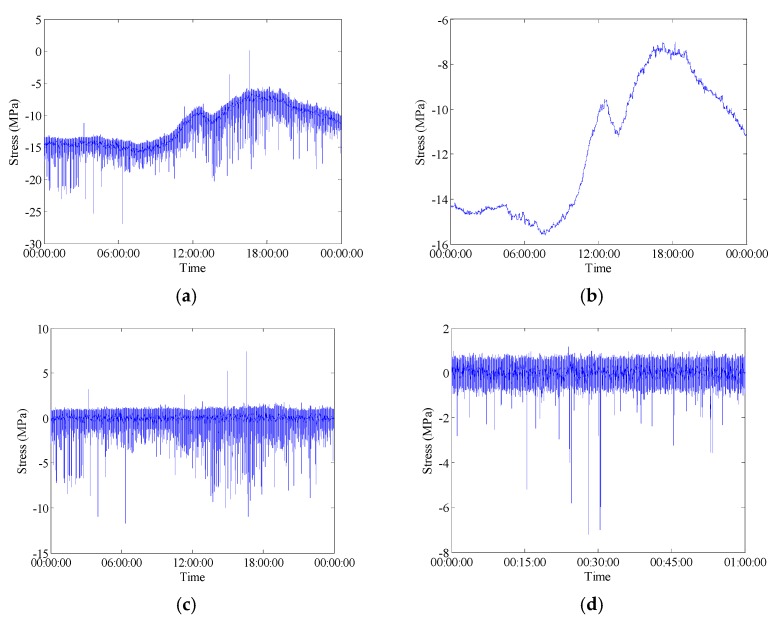

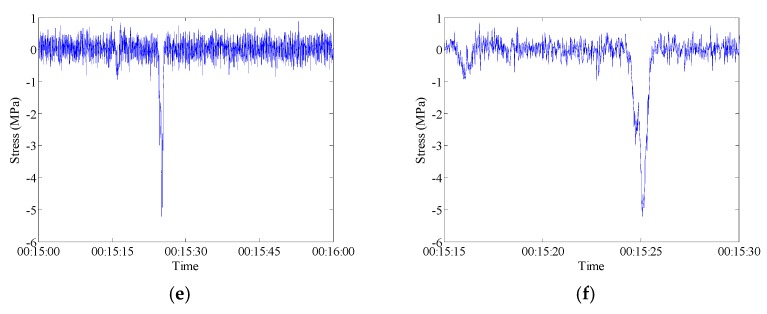
Raw data measured by FBG-MS-NS-2-3: (**a**) Stress time history; (**b**) Temperature time history; (**c**) Live-load induced stress time history; (**d**) Live-load induced stress time history in 1 h; (**e**) Live-load induced stress time history in 1 min; (**f**) Live-load induced stress time history in 15 s.

**Figure 9:**
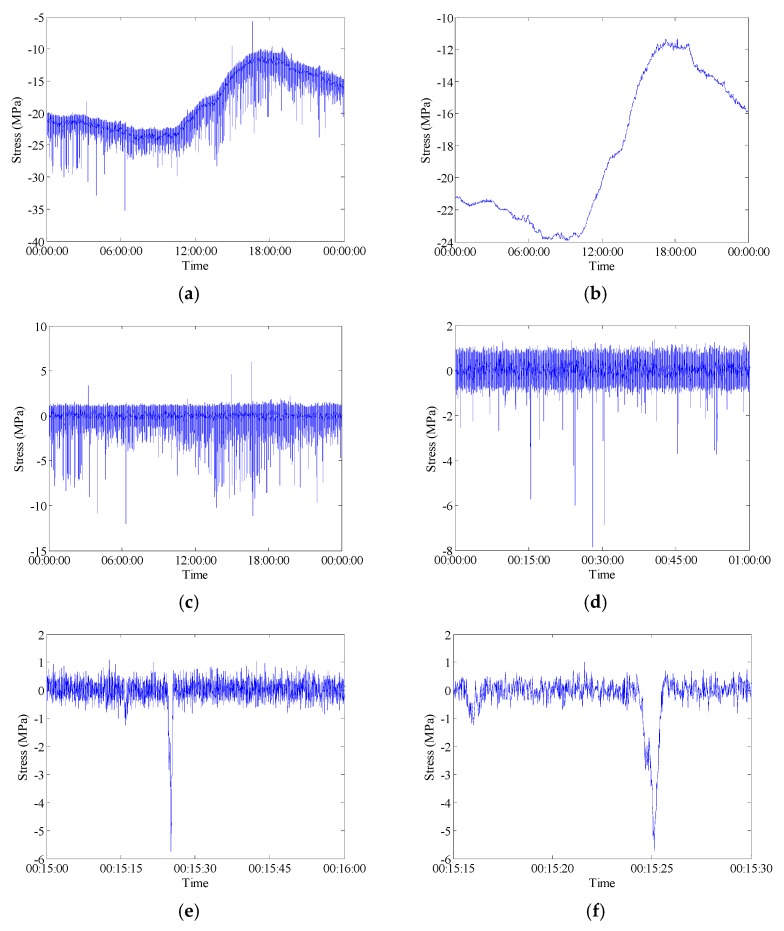
Raw data measured by FBG-MS-NS-2-2: (**a**) Stress time history; (**b**) Temperature time history; (**c**) Live-load induced history (**d**) Live-load induced stress time history in 1 h; (**e**) Live-load induced stress time history in 1 min; (**f**) Live-load induced stress time history in 15 s.

In general, the in-service strain monitoring data acquired from the sensor deployed on a specific position of the bridge are composed of the live-load and temperature induced components. As described in literature [2], there are trend ingredients (low-frequency components) in the stress time histories which can be attributed to be the daily cycle effect of temperature variation. In order to examine the percentage of stress caused by different kinds of loading such as the live-load and temperature, the wavelet multi-resolution analysis approach is used to decompose the raw data into high-frequency and low-frequency components. Wavelet multi-resolution analysis allows a decomposition of the signal into various resolution scales: the data with coarse resolution contain the information about low-frequency components and the data with fine resolution contain the information about high-frequency components [34]. The measured strain signals can be decomposed into approximations and details (i.e., high-frequency and low-frequency components) at various levels. For each level, the high-frequency part (details) is separated and the remaining low-frequency part (approximations) is transferred into the next level of decomposition. Through the wavelet multi-resolution analysis, the strain component attributable to temperature effect can be obtained from the lowest-frequency part in the wavelet transform domain. Figure 8 and Figure 9 illustrate the wavelet-based decomposed stress time histories for the selected FBG strain sensors. In these time histories, the low-frequency parts of 12-level decomposition of stress data represent the reconstructed temperature-induced stress. A comparison analysis between the initial stresses and the temperature-induced stresses indicates that the temperature-induced stress time histories have the same variation tendency and periodic characteristic in one day. The temperature-induced stress takes a major part of the total stress which means that the main stress change during the bridge operation stage is caused by the temperature effect. Figure 8 and Figure 9 also show that the high-frequency parts of 12-level decomposition of stress data induced by live load. The stress histories induced by live load are presented at different time scales (1 day, 1 h, 1 min and 15 s) to show the nature of the signal.

## 3. Probabilistic Modeling of Stress Spectrum

### 3.1. Multimodal Probabilistic Modeling Method

The stress spectrum presents a multi-modal characteristic and its probability density function (PDF) is referred to a mixture density which can be expressed as a weighted sum of different PDFs. The finite mixture distribution model is a combination of two or more PDFs and is usually applied for modeling the PDF of the complex probability distributions [35]. Based on a finite set of PDFs *f*_1_(**x**), *f*_2_(**x**), …, *f*_n_(**x**) and the corresponding weights *w*_1_, *w*_2_, …, *w*_n_ which satisfy more than zero and sum equals one, the basic structure of finite mixture distribution can be written as:

(2) $$f(x|n,w,\theta )=\sum_{l=1}^{n}w_{l}f_{l}(x{|\theta }_{l})$$

where *f*(**x**|*n*,**w**,**θ**) is a target mixture density, *f_l_*(**x**|**θ***_l_*) is a given parametric family of predictive component densities. The estimated parameters of the mixture distribution modeling for stress spectrum includes the number of components or groups, *n*, the mixture weights of components, *w_l_* and the component parameters of each component **θ***_l_*.

In this study, a total of three finite mixture distributions are chosen as the predictive mixture distribution model, i.e., the finite mixed normal distributions, the finite mixed lognormal distributions and the finite mixed Weibull distributions. These distributions can be expressed, respectively, as:

Finite mixed normal distributions:

(3) $$f(x|c,w,\theta )=\sum_{l=1}^{c}w_{l}\frac{1}{\sqrt{2\pi }}\exp\left\{-\frac{1}{2}\frac{{\left(x-\mu_{l}\right)}^{2}}{\sigma_{l}^{2}}\right\}$$

Finite mixed lognormal distributions:

(4) $$f(x|c,w,\theta )=\sum_{l=1}^{c}w_{l}\frac{1}{\sqrt{2\pi }\sigma_{l}x}\exp\left\{-\frac{1}{2}\frac{{\left(\ln(x)-\mu_{l}\right)}^{2}}{\sigma_{l}^{2}}\right\}$$

Finite mixed Weibull distributions:

(5) $$f(x|c,w,\theta )=\sum_{l=1}^{c}w_{l}\frac{\gamma_{l}}{\eta_{l}}{\left(\frac{x}{\eta_{l}}\right)}^{\gamma_{l}-1}\exp\left\{-{\left(\frac{x}{\eta_{l}}\right)}^{\gamma_{l}}\right\}$$

where *μ_l_* and *σ_l_* are the parameters of mean values and standard deviations in normal mixed distributions and lognormal mixed distributions; *γ_l_* and *η_l_* are the shape parameter and scale parameter of Weibull mixed distributions. The unknown parameters in mixture distribution models will be determined by a proposed genetic algorithm (GA)-based parameter estimation method.

GA, proposed by Holland in 1975 [36], is a stochastic algorithm for handling optimization problems which has been widely applied to a variety of problems from fields that include mathematics, civil engineering and astronautics [37]. GA is an optimization process which through selection and modification of individual solutions to successive approach to the optimal solution. This optimization process is similar to the theory of evolution. Like the feature of biological evolution, GA also possesses the advantage enable it to search global area and prevent it from falling to a local optimum. Therefore, GA is especially suitable for the complex and high-dimension optimization problem and it can provide accurate results and less computation time in parameter estimation of finite mixture distribution.

The establishment of a fitness function *T* is the crucial part of GA. Assuming that we have data **x** = [*x_1_*, *x_2_*, *x_3_*, …, *x_n_*]^T^, it is obvious that the closer between the model and the measured data distribution, the larger value of the maximum likelihood function. Thus, the following equation is used as the fitness function:

(6) $$T=f(x|\theta )=f(x_{1}|\theta )\times f(x_{2}|\theta )\times \;\ldots \times f(x_{n}|\theta )$$

To consider if the stress spectra match the selected statistical distribution models, the candidate distribution models with different components need to be compared and then selected based on the fit performance. In this study, the Bayesian information criterion (BIC) is used to determine the number of components and choose an optimal model of the stress spectrum.

BIC, developed by Schwarz, is also based on the likelihood function [38], which is usually defined as:

(7) BIC=ln(n)k−2ln(L)

where *L* is the maximized value of the likelihood function for the models, *k* is a penalty which is the number of estimated parameters in the model and *n* is the number of data points in **x**. The model with the lowest BIC value is preferred.

### 3.2. Modeling of Stress Spectrum Using Monitoring Data

**Figure 10:**
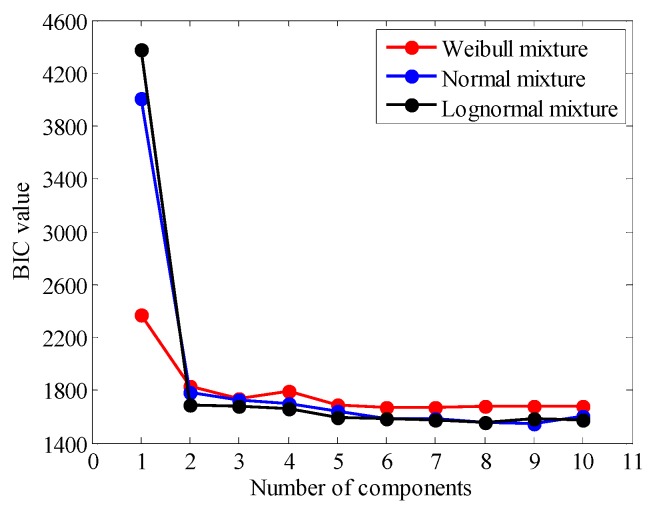
BIC values of mixture distribution of stress range.

**Figure 11:**
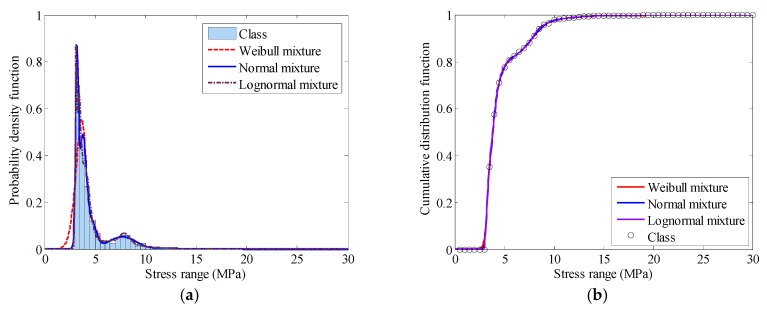
Distribution function of stress range: (**a**) Finite mixture PDFs; (**b**) Finite mixture CDFs.

The stress range data of sensor FBG-MS-NS-2-3 are selected to conduct the probabilistic modeling analysis. Three kinds of finite mixture distributions (normal mixture, lognormal mixture and Weibull mixture) are employed to model the finite mixed PDF and cumulative distribution density (CDF) for the stress range data. The corresponding estimated parameters of different finite mixture distribution models are calculated by the proposed GA-based parameter estimation method. The optimal model is chosen according to the values of BIC. Figure 10 shows the variation of the BIC values with different numbers of components of three finite mixture distribution models. Figure 11 illustrates the PDFs and CDFs of three kinds of finite mixture distributions for the stress range data of the selected FBG sensor. According to the selection criterion, the best model of the stress range distribution is determined with the lowest BIC value. It is found from Figure 10 that the BIC values for three models converge rapidly from two components. The mixture Weibull distribution reaches stable with five components, the mixture normal distribution reaches stable with nine components and the mixture lognormal distribution reaches stable with eight components. Among three distributions, the mixture normal distribution results in the lowest BIC value and it is therefore rational to choose the mixture normal distribution with nine components as the optimal probability distribution of the stress range. The estimated mixture parameters of each component distribution are listed in Table 1.

## 4. Statistical Analysis of Stress Concentration Factor

The stress concentration factor (SCF) can be calculated by dividing the hot spot stress *σ*_hot_ by the nominal stress *σ*_nom_ according to:

(8) $$\mathrm{SCF}=\frac{\sigma_{\mathrm{hot}}}{\sigma_{\mathrm{nom}}}$$

The hot spot stress, resulting from the local stress concentration due to geometric irregularity and discontinuity at the welded joint, is difficult to determine due to the extremely complicated stress distribution in the vicinity of the weld toe. It is commonly obtained by numerical simulations or experimental measurements. According to the IIW specification, the welded joints can be classified into three types (a, b and c) according to the location of hot spot and the weld pattern. In this study, the welded joint adjacent to sensor FBG-MS-NS-2-3 is chosen for case study, which belongs to type b. The hot spot stress of the welded joint is derived based on the stress at the location 5 mm away from the weld toe, *σ*_5mm_ measured by sensor FBG-MS-NS-2-1 and the stress at the location 15 mm away from the weld toe, *σ*_15mm_ measured by sensor FBG-MS-NS-2-2. According to the extrapolation method for type b, it can be calculated by [31]:

(9) $$\sigma_{\mathrm{hot}}=1.5\sigma_{5\mathrm{mm}}-0.5\sigma_{15\mathrm{mm}}$$

**Figure 12:**
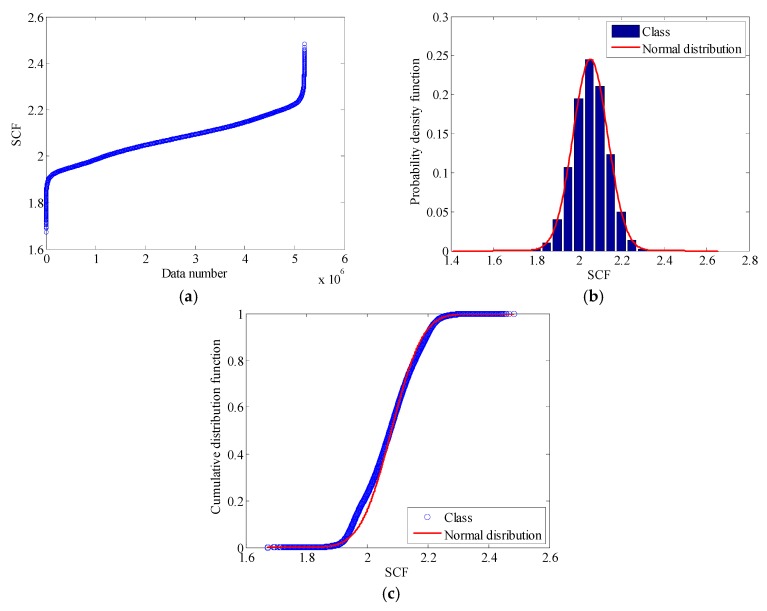
Stochastic property of SCF: (**a**) SCF data in an ascending order; (**b**) PDF of SCF; (**c**) CDF of SCF.

The stress at the location of sensor FBG-MS-NS-2-3 is regarded as the nominal stress and then the SCF can be easily determined as the quotient of hot spot stress and nominal stress according to Equations (8) and (9). Figure 12a illustrates the obtained SCF values presented in an ascending order. The maximum value of the SCF is 2.501 and the minimum value of the SCF is 1.655. Usually, the experimental determination of the SCF is a complicated process considering various factors and uncertainties and the SCF has a nature of randomness. In this study, the SCF is presumed to be a random variable following a normal distribution and the fitted PDF and CDF of the SCF are illustrated in Figure 12b,c. The calculated statistical properties of the SCF are listed in Table 2.

The Kolmogorov-Smirnov (K-S) goodness-of-fit test is a common used nonparametric test approach and usually applied to examine whether or not the observations follow a specified probability distribution [38]. Let *F*_0_(*x*) denotes a specified theoretical continuous CDF, *F*_n_(*x*) is the empirical distribution function for the observations. The test criterion is the maximum absolute difference *D*_n_ between *F*_n_(*x*) and *F*_0_(*x*), which is defined as:

(10) $$D_{n}=\underset{x}{\sup}\left|F_{n}(x)-F_{0}(x)\right|$$

where sup denotes supremum. If *D*_n_ is less than the critical value, the null hypothesis is not rejected and vice versa [39]. In this study, the null hypothesis is that the SCF obeys a normal distribution. The K-S test process is carried out with the aid of the software Matlab by the toolbox function ‘kstest’. For a significance level of 0.05, the SCF conforms to a normal distribution with a mean value of 2.082 in accordance with the K-S test result.

## 5. Conclusions

In this study, an FBG-based field stress monitoring approach was proposed and instrumented on the welded joints of an orthotropic steel bridge crossing the Beijing-Hangzhou Grand Canal located in Hangzhou, China. A total of 64 FBG sensors were deployed on the positions of rib-to-deck and rib-to-diagram welded joints at the mid-span and quarter span sections. The local stress behaviors of the welded joints induced by the live load and temperature effect were measured during the bridge construction and operation periods. The raw strain monitoring data were analyzed with the aid of a wavelet multi-resolution analysis approach. In addition, the multiple characteristic of the rainflow counted stress spectrum was modeled by the method of finite mixture distribution together with a proposed GA-based parameter estimation approach. The mixture normal distribution was determined to be used to model the stress spectrum with the lowest BIC value. Meanwhile, the stochastic property of the SCF was investigated by use of the monitoring data and testified to follow a normal distribution with a mean value of 2.082.

## Figures and Tables

**Table 1:** Estimated mixture parameters of component distributions.

Distribution	Estimated Parameters		
	Weight (*w_l_*)	Mean Value (*μ_l_*)	Standard Deviation (*σ_l_*)
**Mixed normal distribution**	0.18017	7.70356	1.37439
	0.40859	3.77575	0.34666
	0.26359	3.21300	0.14166
	0.00180	18.50252	1.54 × 10^−7^
	0.00180	27.02115	9.11 × 10^−8^
	0.00476	5.51921	0.00440
	0.12739	4.659781	0.45840
	0.00180	15.72309	5.68 × 10^−7^
	0.01005	12.36552	0.91614
**Mixed lognormal distribution**	0.04765	2.07228	0.06052
	0.23927	1.22655	0.05268
	0.18456	1.94656	0.30865
	0.14179	1.13577	0.02487
	0.38311	1.39491	0.11173
	0.00179	2.91790	4.16 × 10^−9^
	0.00179	3.29662	5.86 × 10^−7^
	4.58 × 10^−7^	2.06963	1.48652
**Mixed Weibull distribution**	0.00759	19.3709	2.92343
	0.63222	3.70731	8.32196
	0.02979	10.79137	4.81138
	0.16668	7.98040	6.24103
	0.16369	4.69135	8.37585

**Table 2:** Statistical properties of SCF.

Maximum SCF	Minimum SCF	Mean Value	Standard Deviation	COV
2.501	1.655	2.082	0.081	0.006
